# Localized micro- and nano-scale remodelling in the diabetic aorta

**DOI:** 10.1016/j.actbio.2014.07.001

**Published:** 2014-11

**Authors:** R. Akhtar, J.K. Cruickshank, X. Zhao, L.A. Walton, N.J. Gardiner, S.D. Barrett, H.K. Graham, B. Derby, M.J. Sherratt

**Affiliations:** a Centre for Materials and Structures, School of Engineering, University of Liverpool, Liverpool L69 3GH, UK; b Diabetes & Cardiovascular Medicine, Nutritional Sciences Division, King’s College London, Franklin Wilkins Building, 150 Stamford Street, London SE1 9NH, UK; c School of Materials, Grosvenor St, The University of Manchester, Manchester M1 7HS, UK; d Institute of Cardiovascular Sciences, Faculty of Medical and Human Sciences, The University of Manchester, 46 Grafton Street, Manchester M13 9NT, UK; e Faculty of Life Sciences, AV Hill Building, Oxford Road, The University of Manchester, Manchester M13 9PT, UK; f Surface Science Research Centre, Department of Physics, University of Liverpool, Liverpool, UK; g Institute of Inflammation and Repair, Manchester Academic and Health Sciences Centre, Stopford Building, The University of Manchester, Oxford Road, Manchester M13 9PT, UK

**Keywords:** Arterial stiffening, Fibrillin microfibrils, Type 1 diabetes, Extracellular matrix, Mechanical properties

## Abstract

Diabetes is strongly associated with cardiovascular disease, but the mechanisms, structural and biomechanical consequences of aberrant blood vessel remodelling remain poorly defined. Using an experimental (streptozotocin, STZ) rat model of diabetes, we hypothesized that diabetes enhances extracellular protease activity in the aorta and induces morphological, compositional and localized micromechanical tissue remodelling. We found that the medial aortic layer underwent significant thickening in diabetic animals but without significant changes in collagen or elastin (abundance). Scanning acoustic microscopy demonstrated that such tissue remodelling was associated with a significant decrease in acoustic wave speed (an indicator of reduced material stiffness) in the inter-lamellar spaces of the vessel wall. This index of decreased stiffness was also linked to increased extracellular protease activity (assessed by semi-quantitative in situ gelatin zymography). Such a proteolytically active environment may affect the macromolecular structure of long-lived extracellular matrix molecules. To test this hypothesis, we also characterized the effects of diabetes on the ultrastructure of an important elastic fibre component: the fibrillin microfibril. Using size exclusion chromatography and atomic force microscopy, we isolated and imaged microfibrils from both healthy and diabetic aortas. Microfibrils derived from diabetic tissues were fragmented, morphologically disrupted and weakened (as assessed following molecular combing). These structural and functional abnormalities were not replicated by in vitro glycation. Our data suggest that proteolysis may be a key driver of localized mechanical change in the inter-lamellar space of diabetic rat aortas and that structural proteins (such as fibrillin microfbrils) may be biomarkers of diabetes induced damage.

## Graphical abstract

**Figure:**
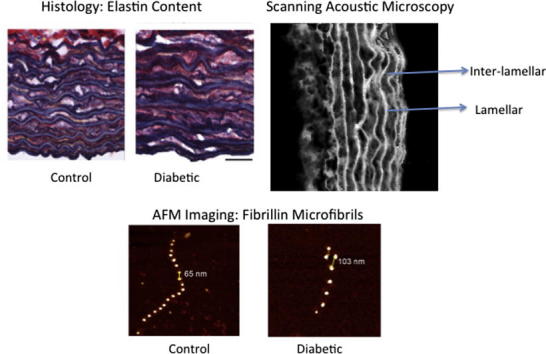

## 1 Introduction

Diabetes is one of the most common non-communicable diseases in the world, with an estimated 382 million people affected worldwide in 2013 [1]. Whether as type 1 or type 2, its major outcomes, or health-related events leading to illness or death, are cardiovascular [2], resulting in reduced life expectancy and greatly increased healthcare costs. In both types, vascular dysfunction occurs early in the disease process [3], [4], [5]. The structural and biomechanical alterations in diabetic macro- and micro-vasculature are complex and the mechanisms remain poorly understood [2], [6], [7]. A better understanding of the processes driving vascular remodelling in diabetes should help develop new therapies [2].

Impaired biomechanical function of the diabetic aorta is generally attributed to changes in the extracellular matrix (ECM), notably in collagen abundance. Most studies suggest that collagen fibrosis causes increased vessel stiffness in the diabetic aorta [6], [8], [9] but there is a lack of consensus in the literature. For example, not all studies have reported increased collagen content in diabetes [10], [11]. Potential mechanisms which underpin this ECM remodelling and hence vessel stiffening include matrix metalloproteinase (MMP) driven catabolic pathways [12], [13], the accumulation of advanced glycation end-product (AGE) cross-links [6], [8] and aberrant transforming growth factor-β (TGF-β) signalling [14]. This latter mechanism may be initiated by disruption of fibrillin microfibril based TGF-β sequestration, as is evident in the profound aortic remodelling which characterizes the vessel prior to rupture in Marfan syndrome, a congenital disease that compromises the mechanical integrity of connective tissues, particularly the aorta [15], [16].

The contribution that micromechanical mapping can make to identifying local vessel stiffening within the vessel wall was highlighted in an earlier review [17] Using scanning acoustic microscopy (SAM), we have previously demonstrated that increased tissue acoustic wave speed (and hence increased stiffness) was localized to medial inter-lamellar regions in both ageing sheep [18] and Cardiotrophin-1 (CT-1) treated rat aortas [19]. In this study, we have used SAM with conventional histology and semi-quantitative in situ zymography to test our first hypothesis that experimental type 1 diabetes would induce morphological, compositional and localized micromechanical remodelling in the aorta associated with increased protease activity.

The lack of consensus regarding structural changes in the diabetic aorta may be due, in part, to the inability of conventional light microscopy to characterize the changing composition and/or macromolecular structure of long-lived ECM proteins [20], [21], [22]. Fibrillar collagens and elastic fibres are complex macromolecular assemblies whose function may be impaired, without affecting their global charge distribution or epitope availability and hence their detection, by histological or immunohistochemical techniques. Fibrillin microfibrils, as key elastic fibre components, play a central role in the pathogenesis of Marfan syndrome. The longevity and well-characterized structure of these microfibrils make them potential structural biomarkers of aberrant tissue remodelling and their role in Marfan syndrome suggests that in situ microfibril damage may be a key trigger for further inflammatory events [16], [23]. In this study therefore we have also employed atomic force microscopy (AFM) and molecular combing [24] to test a second hypothesis that acute diabetes will compromise the ultrastructure and hence extensibility of isolated aortic fibrillin microfibrils [25], [26].

## 2 Materials and methods

### 2.1 Animals and tissues

All procedures accorded to the UK Animals (Scientific Procedures) Act 1986 and the University of Manchester ethical review process. Type 1 diabetes was induced in adult male Wistar rats (Charles River, Kent, UK; 250–300 g; *n* = 9) by a single intraperitoneal injection of streptozotocin (STZ: Sigma Aldrich, Poole, Dorset, UK), freshly dissolved in normal saline, at a dose of 55 mg kg^–1^ [27]. Hyperglycaemia was confirmed (>15 mmol l^–1^) three days following the STZ injection and at the end of the experiment (see Table 1). Experimental and control animals were group housed in Double Decker Rat Housing ICV cages (Tecniplast, Kettering, UK) for 56 ± 0 and 56 ± 1 day, respectively, after which time they were killed by anaesthetic overdose (isoflurane). The mean start and end weights for the controls were 313 ± 11 g and 517 ± 23 g, respectively. The mean start and end weights for the diabetics were 314 ± 13 g and 352 ± 18 g, respectively. The mean blood glucose for the controls was 10.5 ± 0.9 mmol l^–1^. These values are expressed as means ± standard error of the mean (SEM).

**Table 1:** Body weight (start and end weights) and end blood glucose parameters for the Wistar rats. Note all readings for the diabetic rats were higher than the upper limit of detection for the glucose meter, i.e. >27.8 mmol l^–1^.

Group	Start weight (g)	End weight (g)	Blood glucose (mmol l^–1^)
Control	342	629	7.3
Control	365	598	8.1
Control	331	545	9.7
Control	322	647	6.6
Control	318	610	13.7
Control	261	429	12.4
Control	299	514	11.9
Control	282	517	12.7
Control	301	524	12.5
Diabetic	320	305	>27.8
Diabetic	341	404	>27.8
Diabetic	333	396	>27.8
Diabetic	350	329	>27.8
Diabetic	357	437	>27.8
Diabetic	329	362	>27.8
Diabetic	247	282	>27.8
Diabetic	261	300	>27.8
Diabetic	290	350	>27.8

The descending thoracic aorta was dissected and either snap-frozen in liquid nitrogen, or prepared for cryosectioning by freezing in optimal cutting temperature (OCT) resin (Sakura Fintek Europe BV, Alphen aan den Rijn, The Netherlands) in pre-cooled isopentane and stored at −80 °C [18].

### 2.2 SAM

Localized changes in tissue acoustic wave speed were measured for hydrated, unfixed aortic cryosections (5 μm thickness) with SAM as previously described using the Multi-Layer Phase Analysis (MLPA) method [28]. Briefly, SAM imaging was conducted on a KSI 2000 microscope (PVA TePla Analytical Systems GmbH; Herborn, Germany) modified with a custom data acquisition and control system. Imaging was conducted at 760 MHz in this study, which provided a spatial resolution of ∼1.3 μm. The acoustic wave speed (*υ_L_*) is related to Young’s modulus (stiffness) by the following equation:

(1) $$\upsilon_{L}=\sqrt{\frac{C_{11}}{\rho }}=\sqrt{\frac{E}{\rho }\left(\frac{1-\nu }{\left(1+\nu )(1-2\nu \right)}\right)}$$

where *ρ* is the mass density (kg m^−3^), and *C*_11_ (Pa) is a component of the elastic stiffness tensor, which can be expressed a function of Young’s modulus (*E*) and Poisson’s ratio (*ν*) [29]. Hence, a higher acoustic wave speed indicates a stiffer material.

**Fig. 1:**
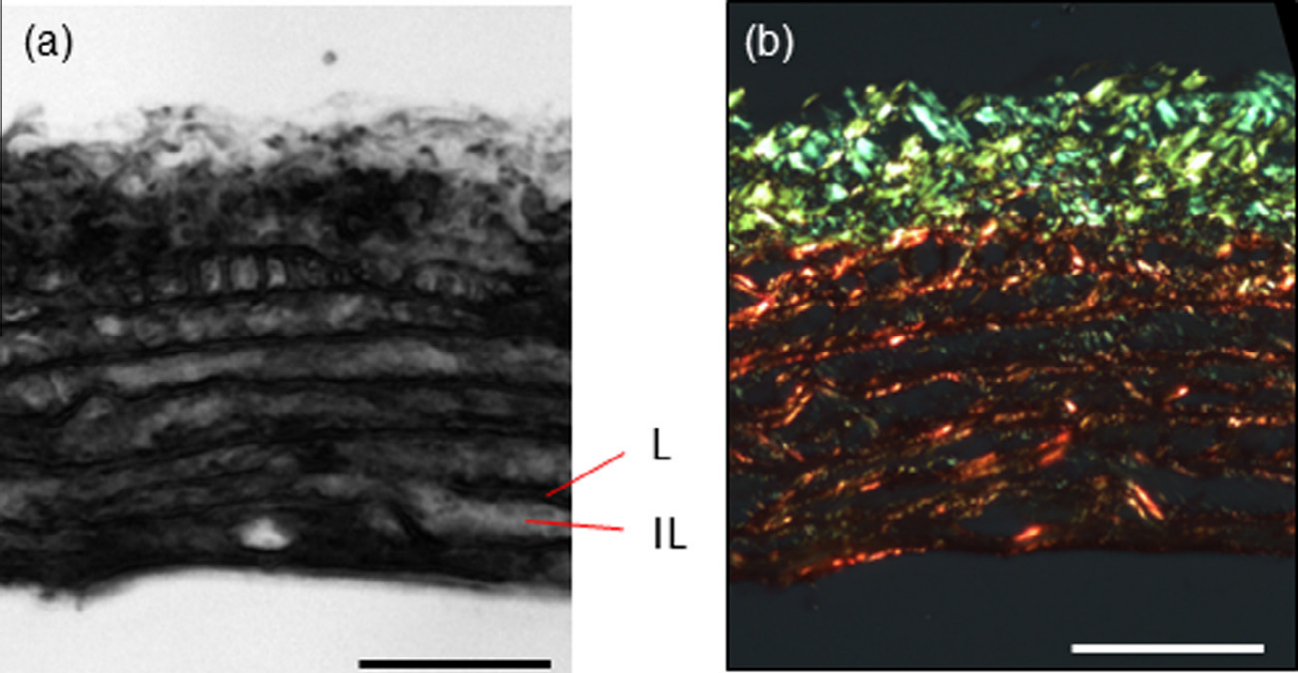
(a) SAM images of rat aorta (200 × 200 μm) prior to any fixation or staining. Lamellar (L) and inter-lamellar (IL) regions of the aorta are highlighted. (b) The same section as shown in (a) following polarized light microscopy of Picro-sirius red (PSR) stained and hence birefringent collagen fibres. Scale bar 50 μm.

The resulting SAM images contain sufficient structural information to allow the acoustic wave speed of the elastic lamellae and inter-lamellar regions of the aortic wall to be measured independently (Fig. 1).

### 2.3 Histological and biochemical analysis

#### 2.3.1 Quantification of collagen and elastin content

The relative fibrillar collagen and elastic fibre content (tissue section area) from control and diabetic rat aortas (*n* = 6 per group) was quantified as previously described [18]. Briefly, 5 μm cryosections were taken from the same animals used for SAM. Collagen content was quantified by obtaining both bright field and circular polarized light images of identical contiguous regions around the aortic circumference. The blue channel from the bright field image was thresholded (which enabled us to exclude voids in the tissue) and total tissue area was measured in pixels. The red channel from the polarized light image of the identical region was thresholded to reveal the collagen positive pixels. Collagen content of each image was then expressed as percentage tissue area. For elastin quantification, the blue channel from the bright field images of Millers stained sections were thresholded to measure the total tissue area (and to exclude voids in the tissue). The red channel was then thresholded to exclusively reveal the blue-black stained elastin fibres. The total elastin positive pixels were expressed as a percentage of total tissue area. Medial thickness (intimal to external elastic lamina of the medial layer) was determined from complete circumference montages of bright field images (at ×100 magnification) of Millers elastic stained cryosections. The images were then thresholded to remove the glass component of the image before medial thickness measurements were taken, therefore only pixels which contained tissue were counted. To ensure that all measurements were taken perpendicular to the intima, curved regions of the aortic wall were straightened using an ImageJ (US National Institutes of Health, Bethesda, Maryland, USA) implementation of a cubic-spline interpolation algorithm [30], [31].

#### 2.3.2 Characterization of ECM protease activity by in situ gelatin zymography

The potential influence of aberrant ECM protease activity on aortic wall remodelling in diabetes was assessed by in situ gelatin zymography of tissue cryosections [32], [33], [34]. Following incubation with an agarose stabilized mixture of the fluorogenic substrate DQ-gelatin and 4′,6-diamidino-2-phenylindole (DAPI) for 1 h at room temperature, areas of gelatin cleavage and DNA localization were visualized by fluorescence microscopy with fluorescein isothiocyanate (FITC) or DAPI filters. Relative gelatinase activity was subsequently quantified for 100 pixel wide regions between the intimal layer and external elastic lamina (*n* = 9; 3 regions from three cryosections) in control and diabetic rat cryosections (*n* = 5 per group).

Low gelling temperature agarose (Sigma Aldrich, Poole, Dorset, UK) was dissolved in 100 ml phosphate-buffered saline (to a final concentration of 10 mg ml^–1^) in a water bath at 80 °C, until a clear solution was obtained. The agarose was stored at 4 °C in air tight vials. DQ gelatin (Invitrogen) was dissolved to a concentration of 1 mg ml^–1^ in dH_2_O and also stored at 4 °C.

The agarose was heated to 60 °C until it had melted and then cooled to 45 °C. The tissue sections, which were 5 μm thick cryosections, were brought to room temperature for 10 min. DAPI was added to the melted agarose to a final concentration of 1 μg ml^–1^ and a diluted DQ gelatin stock solution (1:10) was added to the agarose/DAPI solution; 40 μl of agarose/DAPI/DQ gelatin solution was added onto each tissue section. A coverslip was then placed on the samples to ensure uniformity of film thickness across the specimens. The samples were then incubated at 4 °C in the dark for 18 h (overnight).

Following the incubation, the samples were visualized and imaged immediately. Image analysis was conducted with ImageJ and three regions were analyzed per section, with the green channel used for analysis. Following background subtraction, the mean fluorescence intensity per μm^2^ was then calculated for nine regions (*n* = 3 per section) per animal. Each region measured 20 μm along the axis of the vessel wall and encompassed both the medial and intimal layers.

### 2.4 Extraction and ultrastructural characterization of fibrillin microfibrils

Fibrillin microfibrils were extracted from diabetic and healthy rat aortas by bacterial collagenase digestion, purified by size-exclusion chromatography at physiological pH and adsorbed on poly-l-lysine coated mica substrates [26], [35].

#### 2.4.1 AFM

The fibrillin microfibrils were imaged with AFM (Bruker Multimode and Nanoscope IIIa controller: Bruker AXS, Cambridge, UK) using high aspect ratio etched silicon nitride probes with a nominal spring constant and resonant frequency of 42 N m^−1^ and 300 kHz, respectively (OTESPA probes, Bruker AXS, Cambridge, UK), as previously described [26], [36].

#### 2.4.2 AFM image analysis

Microfibril morphology was characterized by measuring length (number of beads per microfibril) and periodicity (bead-to-bead distance) [37]. A custom routine was written in ImageSXM [38] to allow semi-automated analysis of the AFM images to determine microfibril length and periodicity. Microfibril length (*n* = 100) was quantified as the number of repeats of the characteristic beaded structure.

With this custom ImageSXM routine, the AFM image is loaded with “line-by-line compensation” to reduce the scan line noise inherent in all scanning probe microscopy images. Any artifacts in the image, for example, due to impurities on the sample surface, are identified and the compensation is automatically applied again to improve the discrimination between the microfibril beads and the background. The image is then inverted (to display dark beads on a light background) to enhance contrast. For each microfibril identified in the image, the *x* – *y* position of each bead is recorded to subsequently calculate microfibril periodicity. The number of repeats for each microfibril is also recorded to calculate the length of the microfibril.

#### 2.4.3 Molecular combing of isolated fibrillin microfibrils

The extensibility of isolated fibrillin microfibrils can be characterized using molecular combing [26], a technique which employs a receding meniscus to align and straighten partially adsorbed molecules [39]. We subjected microfibrils extracted from control and diabetic aortas to molecular combing and subsequently quantified their ability to resist an applied surface tension tensile force of ∼4000 pN by measuring microfibril periodicity in AFM height images [26]. Following application of this force, the relative extensibility of the microfibrils can be characterized by comparing the number of extended repeats in control and experimental populations.

#### 2.4.4 In vitro glycation of fibrillin microfibrils

To determine if changes in microfibril structure and extensibility may be induced by glucose-derived cross-linking (glycation), isolated fibrillin microfibrils (derived from the descending aorta of a healthy adult Wistar rat and suspended in column buffer: 400 mM NaCl, 50 mM, Tris–HCl, pH 7.4) were exposed to glucose concentrations of 0, 5 or 100 mmol l^–1^ for 15 days at 37 °C (incubation times and glucose concentrations were adapted from Ref. [40]). The microfibril suspension was divided into three 1 ml aliquots. The first (control) aliquot was supplemented with a bacteriostatic agent (0.01% sodium azide) and incubated for 12 h at 4 °C. The second and third aliquots were dialysed (through Visking tubing: MW cut-off 14 kDa) against 1 l of column buffer supplemented with 0.01% sodium azide, and either 5 mmol l^–1^ or 100 mmol l^–1^ glucose, respectively, for 18 h (with one buffer change) at 4 °C. Subsequently, all aliquots were incubated at 37 °C for 15 days, following which the microfibril structure was characterized from AFM height images of both combed and non-combed AFM samples as previously described (*n* = 500 periodicity measurements per group).

### 2.5 Statistical analysis

Data are expressed as means ± SEM. Standard deviations (SDs) are also reported and indicated as such when reported. The Mann*–*Whitney *U* test was used to compare medial wall thickness, acoustic wave speed, collagen content, elastin content, microfibril periodicity and length in the control and diabetic groups. The Kolmogorov–Smirnov test was used to compare the distribution of microfibril periodicities. The Kruskal*–*Wallis ANOVA test was used for statistical analysis of microfibrillar structural parameters following in vitro exposure to glucose. Where depicted, box and whisker plots represent the interquartile range, with the first and 99th percentile shown with an x symbol. The whiskers represent the upper inner and lower inner fence values.

## 3 Results

### 3.1 Histological analysis

**Fig. 2:**
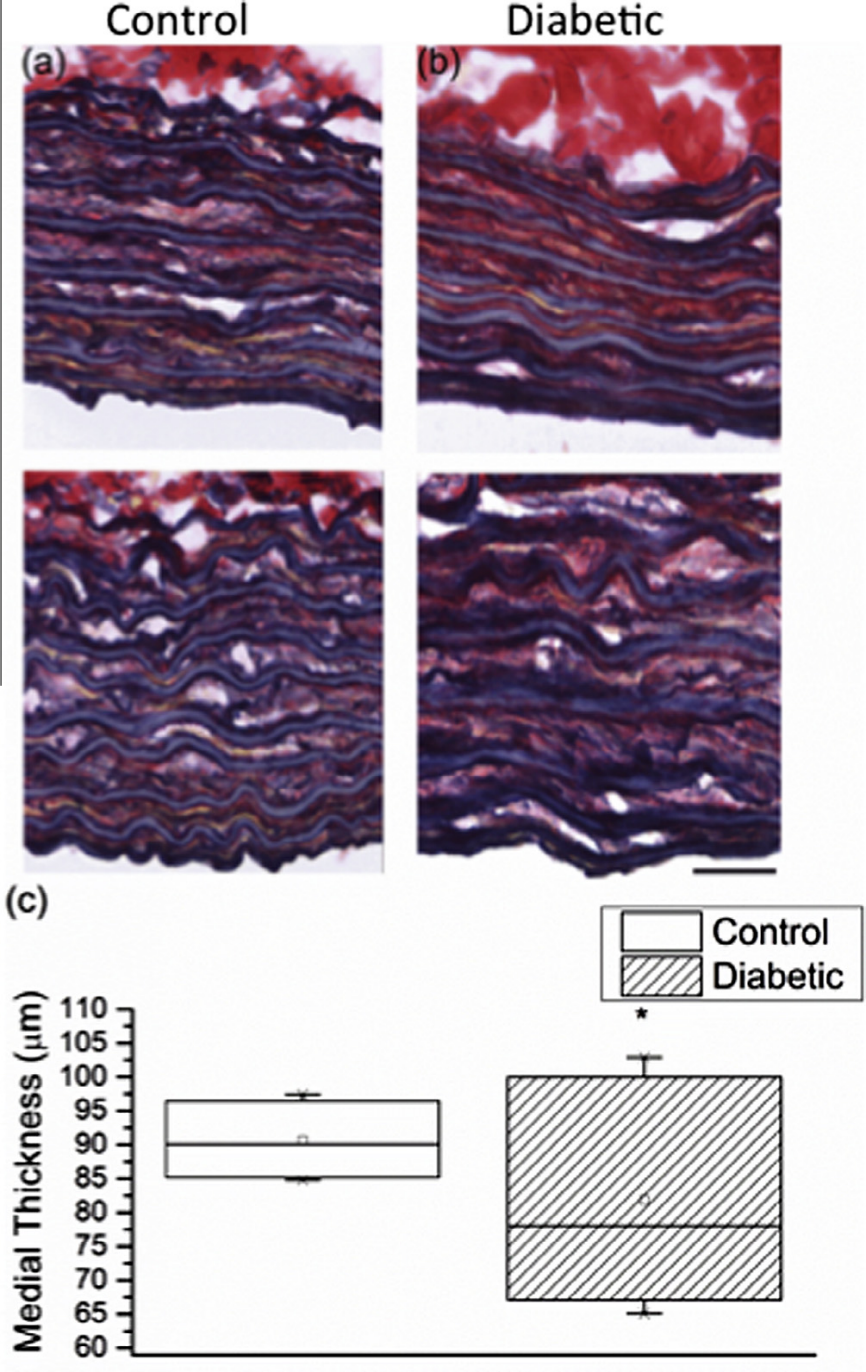
Reduced medial thickness in the diabetic aorta. Typical Millers elastic stained cryosections, used to determine medial thickness, are shown for (a) control and (b) diabetic aortas. The variation in medial thickness across the two groups is evident from the box chart (c). Scale bar: 25 μm.

The aortas of both healthy and diabetic rats were composed of outer adventitial and inner medial layers. In turn the medial layer comprised 7–8 discrete elastic lamellae in both diabetic and control animals (Fig. 2a and b). However, the thickness of the medial layer was significantly lower in the diabetic group (81.9 ± 6.6 μm) as compared to the controls (90.7 ± 2.2 μm) (Mann*–*Whitney *U*-test, *P* < 0.001) (Fig. 2c). Overall, the diabetic medial thickness was much more variable (Fig. 2b), with mean values ranging from 65 to 103 μm compared with 85–97 μm in controls.

**Fig. 3:**
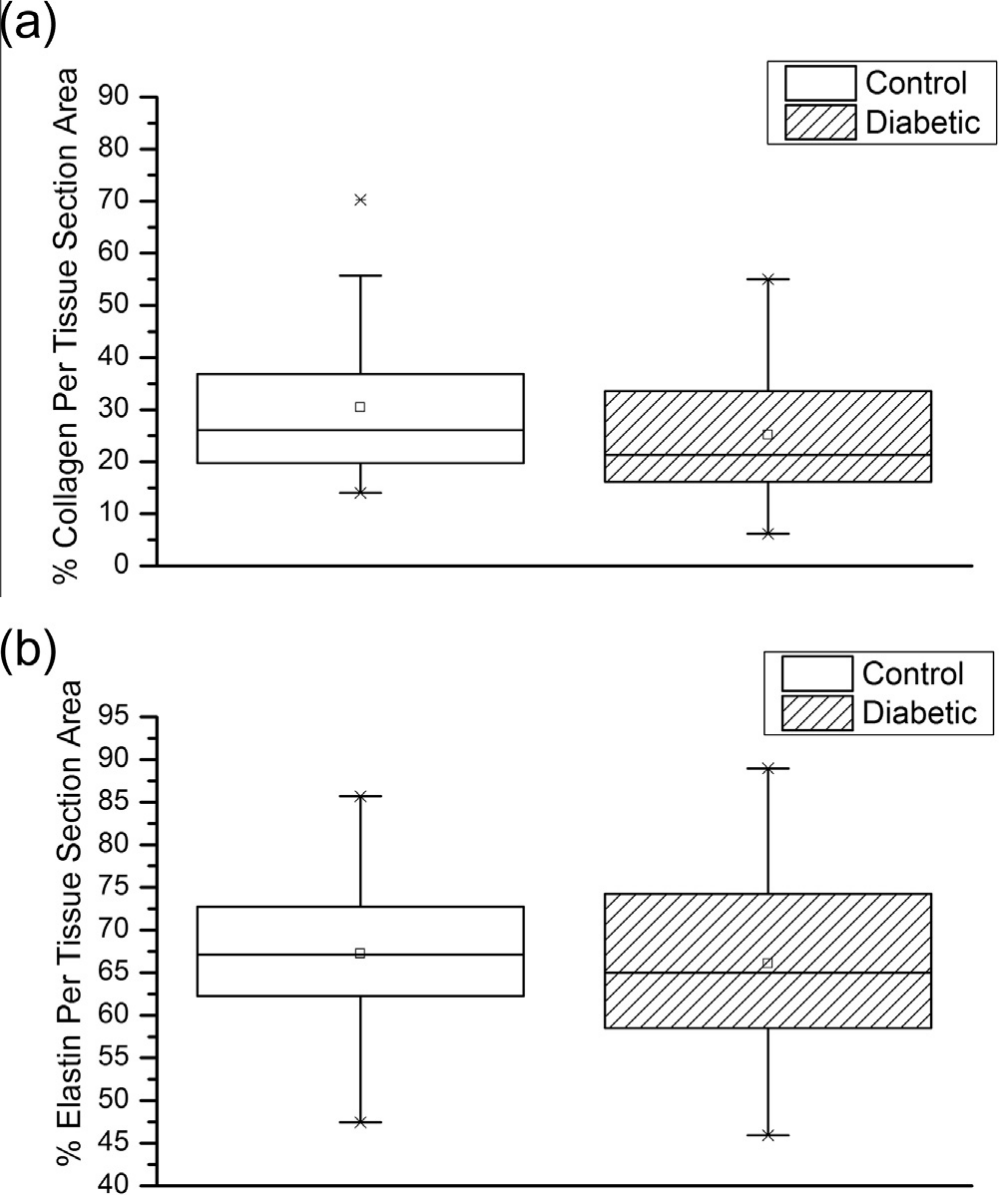
(a) Collagen content: there were 56 and 55 measurements in the control and diabetic group, respectively. (b) Elastin content: there were 57 and 72 measurements in the control and diabetic group, respectively.

Despite these STZ treatment-induced changes in medial layer thickness, there was no significant difference in the abundance of fibrillar collagen between the diabetic (30.4 ± 1.9%) and control aortas (31.0 ± 1.6%) (Mann*–*Whitney *U*-test, *P* = 0.054). Similarly elastin abundance was also unaffected by STZ treatment (control 69.1 ± 2.2%; diabetic 66.1 ± 2.2%) (Mann*–*Whitney *U*-test, *P* = 0.064). (Fig. 3).

### 3.2 Acoustic wave speed

**Fig. 4:**
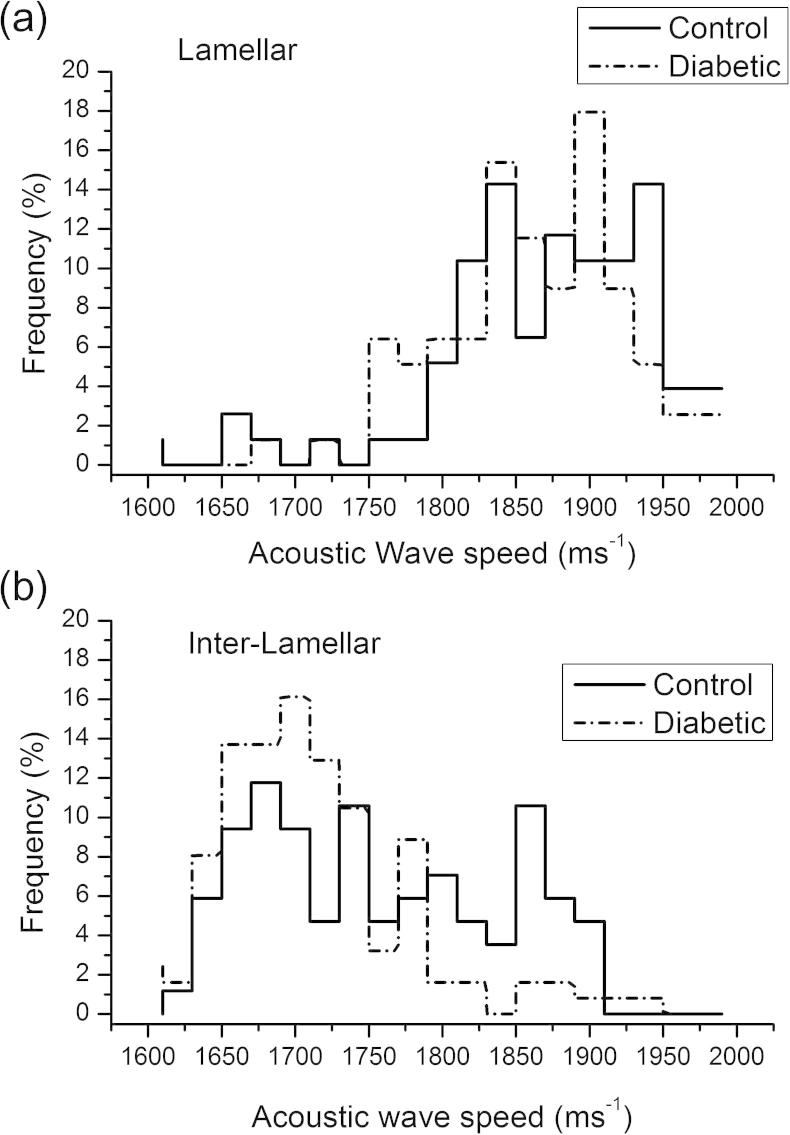
Acoustic wave speed. (a) Acoustic wave speed of elastic lamellae regions. (b) Acoustic wave speed of inter-lamellar regions. Note there is a loss of a distinct peak (at an acoustic wave speed of 1850–1900 m s^−1^) in the diabetic group (*n* = 80 measurements per group).

Diabetes induced changes in vessel morphology were associated with localized reductions in acoustic wave speed. The mean wave speed of the elastic lamellae remained unchanged in diabetic rat aortas compared with controls (control = 1883 ± 6 m s^−1^; diabetic = 1874 ± 7 m s^−1^, Mann*–*Whitney *U*-test, *P* = 0.41), as shown in Fig. 4a, but was significantly reduced (by 34 m s^−1^) in the inter-lamellar regions (Mann*–*Whitney *U*-test, *P* < 0.01) with the wave speed frequency distribution being unimodal as compared to bimodal in the controls (Fig. 4b).

### 3.3 Protease activity

**Fig. 5:**
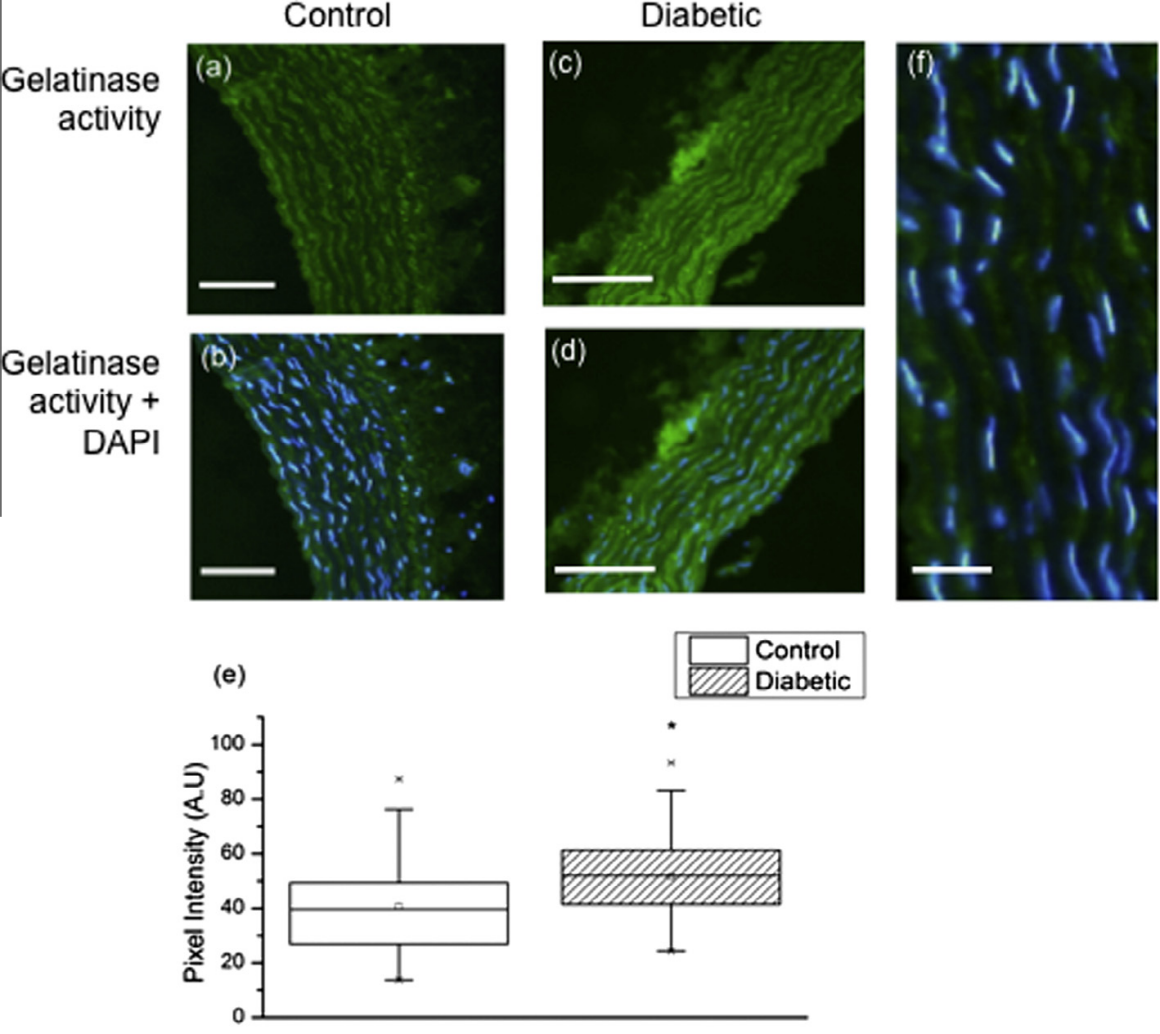
Gelatinase activity. (a)–(d) Florescent micrographs of control (a) and (b), and diabetic (c) and (d) aorta cryosections. Green fluorescence (FITC) indicates areas of gelatinase activity whilst blue fluorescence (DAPI) marks DNA in cell nuclei. (e) Box and whisker plot showing difference in gelatinase activity. (f) This activity is localized to the interlamellar regions which contain vascular smooth muscle cells (as visualized by DAPI). Scales bars 50 μm (a*–*d) and 20 μm (f).

The structure and mechanical properties of large arteries such as the aorta are dominated by the ECM proteins, which in turn are thought to be remodelled in situ primarily by members of a large family of zinc-dependent endopeptidases: the matrix metalloproteinases (MMPs) [41]. These MMPs, which are active in diabetic vessels [12], [42], [43], also act as gelatinases; hence in this study we used in situ gelatin zymography to localize and quantify aortic gelatinase activity in both control and diabetic vessels. Gelatinase activity was concentrated in the inter-lamellar regions in both diabetic and control vessels but was significantly higher in STZ treated animals (*P* *<* 0.0001) (Fig. 5a–d). As shown in Fig. 5e, the mean pixel intensity was higher in the diabetic (51.6 absorbance units: A.U.) compared with the controls (40.6 A.U.) The key gelatinases such as MMPs 2, 3, 9, 12 and 13, which are present in the diabetic aorta, are also known to degrade fibrillin microfibrils [44]; thus we next determined if these important elastic fibre components have the potential to act as structural biomarkers of accumulated damage in diabetic tissues.

### 3.4 Fibrillin microfibril ultrastructure and extensibility

**Fig. 6:**
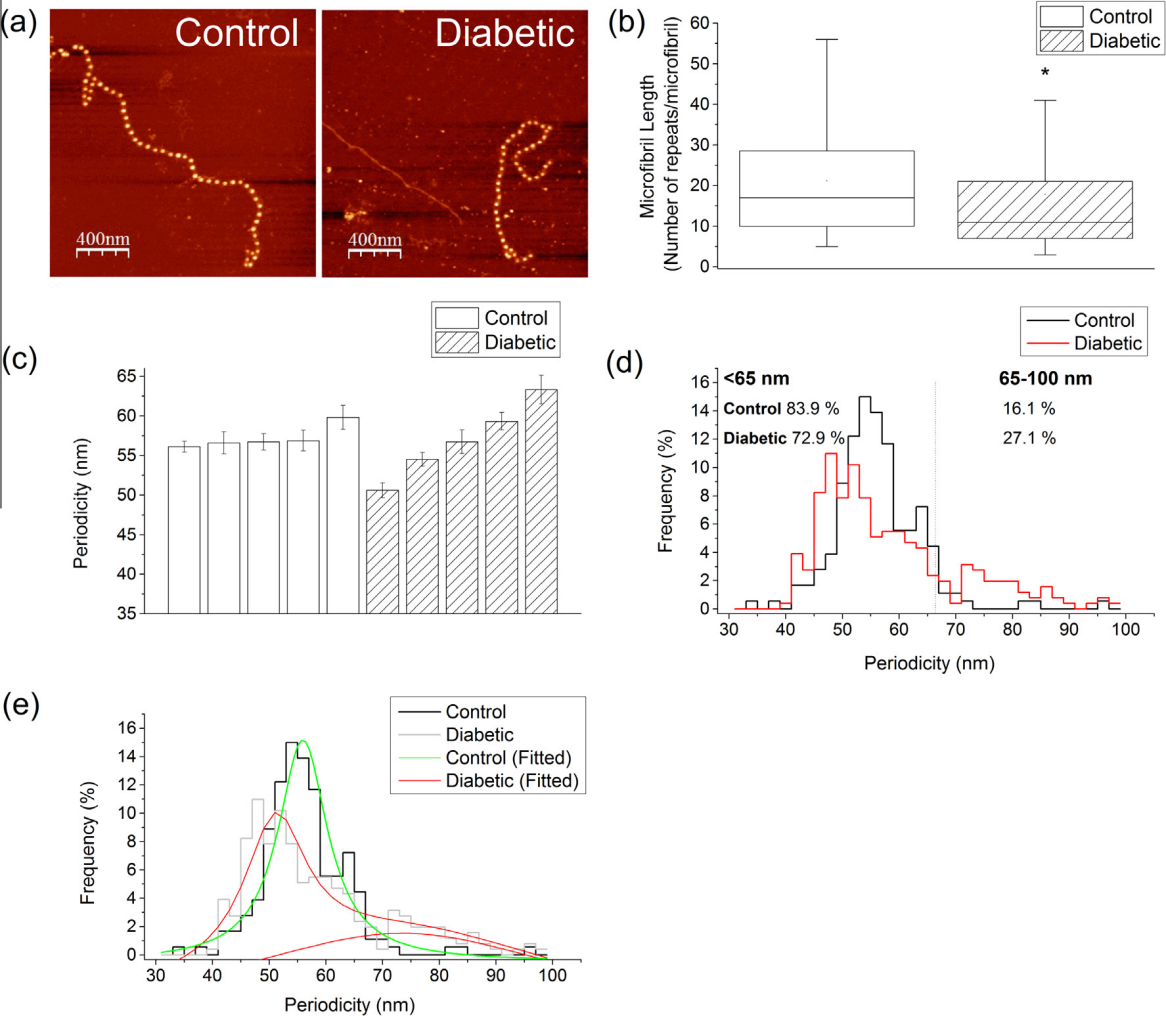
Fibrillin microfibril morphology. (a) Abundant fibrillin microfibrils were isolated from control and diabetic aorta and imaged with AFM. (b) Fibrillin microfibril length (*n* = 100 length measurements per group). (c) Mean microfibril periodicity. Each bar represents an individual animal. 500 individual periodicity measurements were made for each animal. (d) Histogram showing a unimodal distribution in the controls as compared to bi-modal periodicity distribution in the diabetic group. (e) Lorentzian fit of the periodicity histogram data confirming that in the control group the distribution is centred at 56 nm (Lorentzian fit, *R*^2^ = 0.95 nm) whereas in the diabetic group it follows a bi-modal distribution centred at 51 and 73 nm, Lorentzian fit, *R*^2^ = 0.89 nm). Note there are two fitted peaks for the diabetic group (in red).

Mammalian aortas are abundant sources of fibrillin microfibrils. These assemblies form extensive chains with typical repeat distances (periodicities) of 56 nm [26], [45]. Although abundant fibrillin microfibrils were isolated from all arterial tissue samples regardless of disease state (Fig. 6a), microfibrils extracted from diabetic tissue were significantly shorter (Fig. 6b). The control mean length was 21 ± 1 beads as compared to 18 ± 2 beads in the diabetic group (Mann*–*Whitney *U* test, *P* < 0.001). Microfibril length was also less variable in the control group as compared to the diabetic group, as was evident by the lower standard deviation (control SD 13 beads; diabetic SD 19 beads). Furthermore, the diabetic group exhibited an altered periodicity distribution compared to assemblies derived from control tissue (Fig. 6c–e). Overall, mean microfibril periodicity was higher in the diabetic group (control mean = 57.2 ± 0.6 nm; diabetic mean = 59.2 ± 0.8 nm). The two distributions were significantly different (Kolmogorov*–*Smirnoz test, *P* < 0.01). In the diabetic group, 27.1% of microfibrils were extended above 65 nm as compared to 16.1% in the controls. Specifically, the periodicity distribution of control microfibrils was unimodally distributed with a peak centred at 56 nm whereas microfibrils extracted from diabetic tissue were distributed into two populations with resting periodicities 51 and 73 nm. A Lorentzian fit of the periodicity histogram data confirmed that in the control group the distribution is centred at 56 nm (*R*^2^ = 0.95 nm). In contrast, the mean microfibril periodicity in the diabetic group was found to follow a bi-modal distribution centred at 51 and 73 nm, *R*^2^ = 0.89 nm). The fitted data are shown in Fig. 6e.

**Fig. 7:**
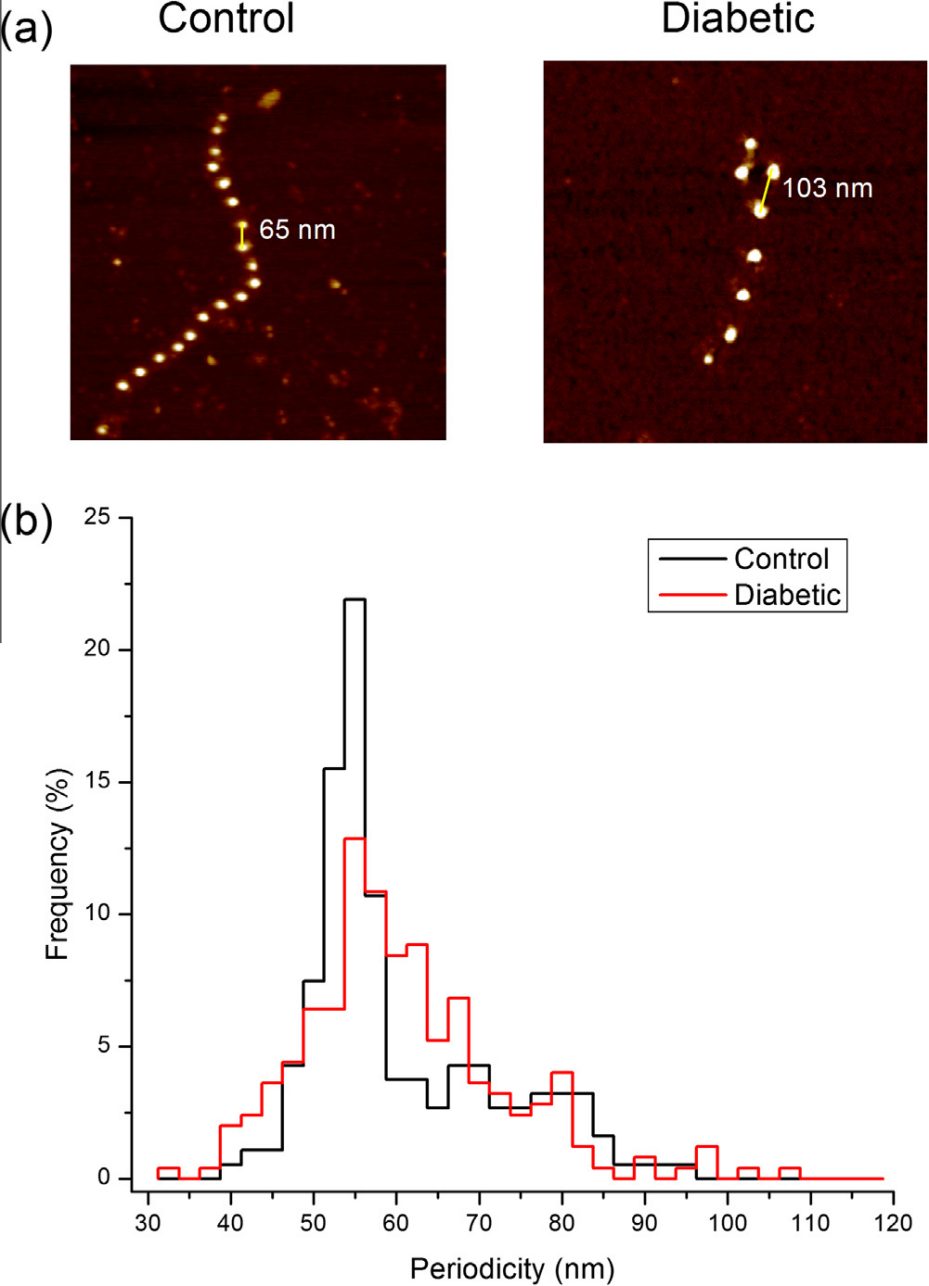
Fibrillin microfibril extensibility: (a) AFM height images (1 × 1 μm) of control and diabetic fibrillin microfibrils subjected to molecular combing. (b) Histogram of microfibril periodicities following molecular combing. 500 individual periodicity measurements were made for each animal.

In addition to their biochemical role in mediating tissue homeostasis, fibrillin microfibrils are required to perform mechanical roles both on their own (in the eye where they suspend the lens and in skin where they intercalate into the dermal*–*epidermal junction) and potentially in combination with elastin, where they reinforce the elastic fibre [26], [46], [47]. Hence in this study, we employed molecular combing to apply a capillary tensile force to partially adsorbed microfibrils (Fig. 7a). Following application of this force significantly more repeats were extended beyond 60 nm in the diabetic as compared with the control populations. Extension beyond 60 nm was observed in only 37% of microfibril repeats within the control population, compared with 50% of the repeats measured in the diabetic population (Kolmogorov–Smirnov test, *P* < 0.05) (Fig. 7b).

### 3.5 Fibrillin microfibril ultrastructure and extensibility following in vitro glycation

**Fig. 8:**
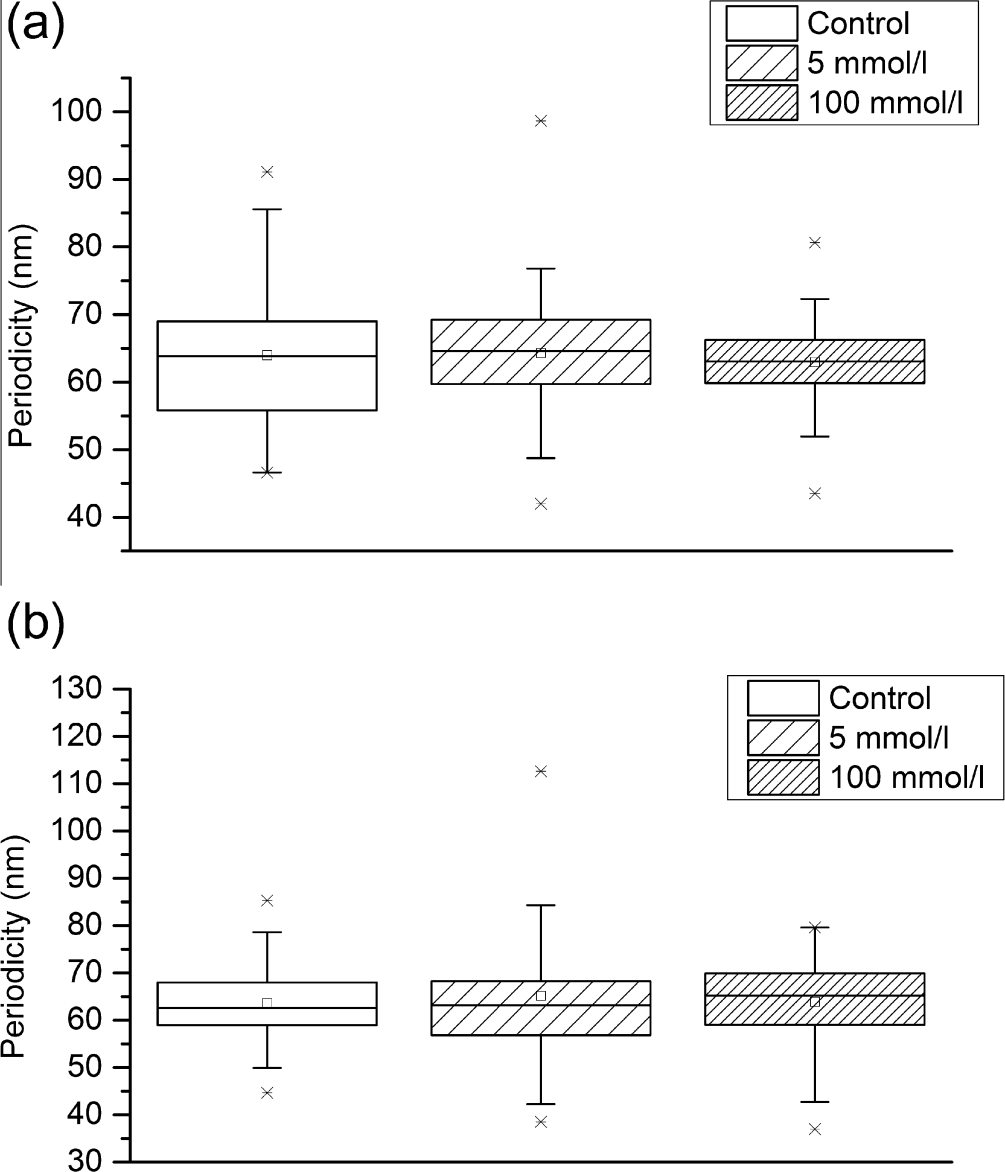
In vitro glycation of fibrillin microfibrils. Box and whisker plot showing periodicity distribution: (a) untensioned periodicity; (b) periodicity following molecular combing. 500 periodicity measurements were made in each group.

ECM components including fibrillar collagens and elastin may accumulate glucose-derived cross-links with both increasing age and diabetes, which may, in turn, affect the molecular and hence macromechanical properties of tissues [48]. As isolated fibrillin monomers are susceptible to glycation [49], in this study we determined whether direct exposure to glucose could recapitulate in vitro the structural and mechanical effects of diabetes on fibrillin microfibril structure and extensibility which we observed in vivo. Isolated microfibrils were exposed to 5 mmol l^–1^ and 100 mmol l^–1^ glucose concentrations at physiological temperatures in vitro. However, despite the prolonged exposure time (more than 2 weeks), we observed no correlation between glucose concentration and microfibril periodicity, as shown in Fig. 8a (control [no glucose] mean = 64.0 ± 1.2 nm; 5 mmol l^–1^ mean = 64.3 nm ± 1.2 nm; 100 mmol l^–1^ mean = 63.0 ± 0.9 nm), Kruskall*–*Wallis ANOVA *P* = 0.45. There was also no significant difference in periodicity between control microfibrils and microfibrils exposed to 100 mmol l^–1^ glucose (ANOVA, *P* = 0.52). Furthermore, there was no correlation between glucose concentration and microfibril periodicity following molecular combing as shown in Fig. 8b (control [no glucose] mean = 63.6 ± 1.1 nm; 5 mmol l^–1^ mean = 65.1 ± 1.8 nm; 100 mmol l^–1^ mean = 63.9 ± 1.0 nm; Kruskal*–*Wallis ANOVA, *P* = 0.63).

## 4 Discussion

In this study, we have utilized one of the most widely used animal models of human disease, where diabetes is induced by selective destruction of the insulin-producing B-cells of the pancreas with a single, rapid injection of STZ [50]. The chronic STZ diabetic rat has been found to mimic many of the chronic complications that are observed in the diabetic human, and has further potential as a model to test new therapeutic approaches for the alleviation of chronic diabetic complications in humans [50]. Due to its association with relatively acute insulin deficiency, the model can also be useful to compare with pathophysiological changes in newly diagnosed Type 1 diabetic patients, i.e. before insulin treatment has begun [51]. Tomlinson et al. [51] have provided a detailed review of functional changes in the cardiovascular system in the STZ model, with reference to pathological chronic diabetes in humans.

The data we report in this study support the hypothesis that experimentally induced diabetes may cause protease-mediated morphological and micro-mechanical remodelling of the ECM. Our findings also suggest that STZ-treatment primarily affects vessel architecture (at multiple lengths scales) rather than molecular composition. Collagen content has previously been reported to increase [6], [8], decrease [10] and remain unchanged [11] in the diabetic aorta. The data presented here support the observations of Salum et al. [11] that diabetes induces loss of medial layer organization and thickness. We further show that the structure and stiffness of the fibrillin microfibril are compromised in diabetic vessels. These structural changes, in turn, have functional implications for the micro-mechanical stiffness of the vessel.

### 4.1 Acoustic wave speed

Gross changes in rat vessel compliance may only become evident in the latter stages of hypertension and at supra-physiological arterial pressures [11]. In common with the pulse wave velocity (PWV) measurements of vessel stiffness as employed by Salum et al. [11], our assessment of acoustic wave speed in the medial layer failed to distinguish between the healthy and diabetic groups. Hence the relative insensitivity of gross mechanical measurement methods may explain the current lack of consensus with regard to mechanical consequences of diabetes in large arteries [8], [52]. However, using SAM to resolve the individual mechanical contributions of discrete vessel sub-structures [18], we identified significant changes in acoustic wave speed that were localized to the inter-lamellar regions of the medial layer. Therefore this technique is able to identify both localized increases in ageing sheep aorta [18] and CT-1 exposed rats [20]), as well as decreases (STZ-treated rats – this study) in the acoustic wave speed of medial inter-lamellar regions.

### 4.2 Protease activity

The adverse influence of diabetes on both the architecture of the elastic fibre system [53] and the molecular structure and mechanical properties of elastin is well established [54] and there is substantial evidence that increased ECM-protease activity may play a major role in mediating structural and hence mechanical remodelling in the diabetic aorta [12], [47]. Given that all three implicated MMPs (−2, −9 and −12), in common with most MMPs, not only act as gelatinases [42] but also degrade fibrillin microfibrils, we used in situ gelatin zymography both to locate protease activity to the inter-lamellar regions of the medial layer and to demonstrate that this activity was increased in diabetic vessels. These data suggest that the ultrastructural and mechanical remodelling which we observe in the isolated microfibrils and the vessel wall may, in part, be driven by activated MMPs. In future work, it would be help to further identify protease classes using in situ elastin zymography.

### 4.3 Fibrillin microfibrils

As fibrillin microfibrils, in common with other ECM structural components, are thought to have a tissue half-life of many years [20], [55], we tested the hypothesis that they may accumulate macromolecular damage in diabetic vessels and hence act as structural biomarkers of disease progression. In normal, healthy connective tissues fibrillin microfibrils have a characteristic periodicity of 56 nm [46]. However, it has been reported that alterations in microfibril periodicity can be induced by heritable mutations in the fibrillion-1 gene (FBN-1) [56], by modification of the in vitro environment such as via calcium chelation [57], [58], reduced salt concentration [59] and also by adsorption to high surface energy substrates [38]. Within the whole organism such genetic modifications can adversely affect microfibril function; specifically, FBN-1 point mutations. Mutations in the fibrillin-1 gene FBN-1 may cause aberrant fibrillin microfibril assembly and hence the profound aortic pathologies which characterize Marfan syndrome [50]. Here we demonstrate that microfibril structure and extensibility [26] may also be compromised in a diseased tissue. These observations support our hypothesis for the potential utility of fibrillin microfibrils as structural biomarkers of tissue remodelling and also suggest that protease mediated remodelling of microfibril structure may promote further remodelling of the tissue because of aberrant downstream TGF-β sequestration by functionally incompetent microfibrils [60]. Finally, our data indicate that glycation events are unlikely to mediate the modification of microfibrils in vivo. Although Atanasova et al. [41] demonstrated that human aortic fibrillin-1 monomers were susceptible to non-enzymatic glycation, in its assembled form many regions of the monomer are shielded from enzymatic action [61] and hence potentially from reaction with glucose. The importance of a quaternary structure in mediating non-enzymatic glycation events has previously been suggested by Slatter et al. [62] for collagen fibrils.

In summary, our study demonstrates that in diabetes there is a profound change in the inter-lamellar regions of the medial layer of the aorta coupled with an altered morphology and reduced extensibility of fibrillin microfibrils. It therefore seems likely that there is an early loss of arterial integrity in diabetes, as suggested by Salum et al. [11], which is not detectable with conventional in vivo or in vitro mechanical testing methods. Further studies will be required to identify the biochemical nature and precise molecular pathology of these structural and micro-mechanical remodelling events.

## Conflict of interest

There are no conflicts of interest to declare.

## Appendix A Figures with essential color discrimination

Certain figures in this article, particularly Figs. 1, 2, 5–7 are difficult to interpret in black and white. The full color images can be found in the on-line version, at http://dx.doi.org/10.1016/j.actbio.2014.07.001.
